# Adolescent depression and adult labor market marginalization: a longitudinal cohort study

**DOI:** 10.1007/s00787-021-01825-3

**Published:** 2021-06-25

**Authors:** Iman Alaie, Anna Philipson, Richard Ssegonja, William E. Copeland, Mia Ramklint, Hannes Bohman, Ulf Jonsson

**Affiliations:** 1 Department of Neuroscience, Child and Adolescent Psychiatry, Uppsala University, Uppsala, Sweden; 2 University Health Care Research Center, Faculty of Medicine and Health, Örebro University, Örebro, Sweden; 3 Department of Public Health and Caring Sciences, Child Health and Parenting (CHAP), Uppsala University, Uppsala, Sweden; 4 Department of Medical Sciences, Respiratory-, Allergy-, and Sleep Research Unit, Uppsala University, Uppsala, Sweden; 5 Department of Psychiatry, Vermont Center for Children, Youth, and Families, University of Vermont, Burlington, USA; 6 Karolinska Institutet Center of Neurodevelopmental Disorders (KIND), Centre for Psychiatry Research, Department of Women’s and Children’s Health, Karolinska Institutet, and Stockholm Health Care Services, Stockholm County Council, Stockholm, Sweden

**Keywords:** Depression, Adolescent, Academic success, Employment, Longitudinal studies

## Abstract

Adolescent depression is linked to adult ill-health and functional impairment, but recent research suggests that individual/contextual factors might account for this association. This study aimed to test whether the clinical heterogeneity of adolescent depression is related to marginalization from the labor market across early to middle adulthood. Data were drawn from the Uppsala Longitudinal Adolescent Depression Study, a community-based cohort initially assessed with structured clinical interviews at age 16–17. The cohort (*n* = 321 depressed; *n* = 218 nondepressed) was followed up after 2+ decades through linkage to nationwide population-based registries. Outcomes included consecutive annual data on unemployment, work disability, social welfare recipiency, and a composite marginalization measure, spanning from age 21 to 40. Longitudinal associations were examined using logistic regression analysis in a generalized estimating equations modeling framework. Subsequent depressive episodes and educational attainment in early adulthood were explored as potential pathways. The results showed that adolescent depression was associated with adult marginalization outcomes, but the strength of association varied across depressed subgroups. Adolescents with persistent depressive disorder had higher odds of all outcomes, including the composite marginalization measure (adjusted OR = 2.0, 95% CI = 1.4–2.7, *p* < 0.001), and this was partially (31%) mediated by subsequent depressive episodes in early adulthood. Exploratory moderation analysis revealed that entry into tertiary education mitigated the association with later marginalization, but only for adolescents with episodic major depression. In conclusion, the risk for future labor market marginalization is elevated among depressed adolescents, particularly those presenting with persistent depressive disorder. Targeted interventions seem crucial to mitigate the long-lasting impact of early-onset depression.

## Introduction

Increasing longitudinal evidence corroborates that depression in adolescence is linked to poor general health [1] and mental ill-health [2] in adulthood. Meta-analytic findings also imply substantial impairments in adult psychosocial functioning among a large proportion of those with adolescent depression [3, 4]. As adolescence is characterized by a rapid cognitive, emotional, and social development, in which role transitions and acquisition of life skills are ever advancing [5], it is important to better understand how and for whom depression may interfere with later adjustment to adult life. Such understanding is central to improving targeted prevention and treatment, but is also highly relevant from a societal perspective insofar as early-onset common mental disorders and even subclinical mental health problems may disrupt future role functioning, including educational aspiration and workforce participation [6–10].

The widespread problem of not being in paid work due to unemployment or work incapacity, sometimes referred to as labor market marginalization (LMM) [11], has been increasingly recognized as a major societal challenge [12], in particular for young people suffering from common mental disorders [13, 14]. Considering that mental ill-health is suggested to be both a determinant and a consequence of unemployment [15], the longer-term effects of adolescent depression on adulthood employability, sickness absenteeism, and overall economic self-sufficiency are arguably of particular concern. Previous epidemiologic research examining the risk of adult unemployment and welfare dependency has been rather inconclusive [3, 4, 16], thereby raising the question of heterogeneity in adolescent depression. Several longitudinal studies have focused on subthreshold depressive symptoms or major depression more generally, albeit with an insufficient clinical differentiation between depressive disorders. Evidence from a long-term follow-up of a birth cohort suggests that the associations of adolescent depression with adverse adult outcomes are mainly attributable to adolescent functioning and early contextual/familial adversities [17]. On the other hand, a recent large-scale study based on hospital-diagnosed depression clearly demonstrates increased risks of poor socioeconomic outcomes, including unemployment and low educational attainment [18]. These findings altogether underscore the need to investigate whether the long-term impairment may differ by the clinical form of adolescent depression.

Although a few pioneering studies using community samples or birth cohorts have looked at adult labor market outcomes following adolescent depression [3, 4], the overall generalizability and comparability of past research efforts are still quite limited, and several important questions remain unanswered. First, with few exceptions [17, 19, 20], most studies have followed up into early adulthood only (e.g., < 30 years of age) [3], thereby leaving the question of the longer-term outcome unanswered. This points to the need of using repeated measurements over extended periods of time. Second, the sole reliance on self-reported outcome measures remains a nontrivial limitation of prior research, due to potential bias and recall failure [21]. Linkage with nationwide population-based registries would partly resolve such issues [22]. Third, with a single exception [23], current literature on adult labor market outcomes associated with adolescent depression spans over domains of unemployment and welfare dependency [3, 4], while less is known about functional impairment in terms of sickness absenteeism. Again, this emerges as an important area of further research in view of overall life prospects following early-onset common mental disorders [13, 14]. Fourth, there is still limited knowledge about early chronic/persistent depression, which is reported to have a particularly poor prognosis [19, 24, 25]. Fifth, there is also the need to address the mechanisms for the labor market outcome, notably the role played by the course of depressive illness and the educational journeys during early adulthood.

In a recently published study [20], we estimated the differences between formerly depressed adolescents and nondepressed peers with respect to earnings across early to middle adulthood. We found that chronic/persistent depression, in particular, was linked to lower adult earnings, yet it is unclear how this loss of productivity is reflected in workforce participation and overall economic self-sufficiency. It could be hypothesized that adolescents with depression in general are at elevated risk of marginalization from the labor market in adulthood, and that those with early chronic/persistent depression may face a particularly increased risk of later adversities [25]. Previous findings show that depression recurrence in early adulthood plays a key role in determining adult outcome [20, 26]; however, it remains to be seen to what extent this also applies to LMM.

While the process of making a successful transition to adulthood depends on a multitude of structural and individual characteristics [27, 28], cross-national comparative data clearly show that young people with tertiary education do much better in the labor market than those with lower education [13]. Comparative research also shows that school-to-work trajectories appear to be quite similar in the United States and several European countries [29, 30], with most people completing education and commencing work by their mid-twenties [13]. Some previous follow-up studies have examined the association between adolescent depression and various educational outcomes, suggesting higher odds of failure to complete secondary school and lower odds of postsecondary entry, as compared with nondepressed adolescents [3]. Indeed, one of our own previous studies found that depressed adolescents were less likely to graduate from college/university by age 30 than their nondepressed peers [31]. Nonetheless, there is a dearth of prospective research simultaneously addressing how educational attainment and adult depression impact the association between adolescent depression and subsequent labor market outcomes. The only exception is a report suggesting that this association may partly be attributable to these potential pathways [16]. While that particular study relied on self-reported depressive symptoms across all follow-up measurements, it still implies that this link should be explored further [16]. As such, it remains unclear whether low educational attainment may be linked to worse long-term prospects on the labor market among those exposed to depression in early life, as compared with their nondepressed peers.

Herein, we attempted to address these unresolved issues using a prospective cohort study spanning over more than 2 decades. The overarching aims were: (a) to analyze longitudinal associations of adolescent depression with subsequent long-term unemployment, work disability, and social welfare recipiency, while adjusting for potential confounders; (b) to test if these associations differ by clinical form of adolescent depression; (c) to test the extent to which depressive episodes in early adulthood may mediate the subsequent labor market outcome, and (d) to explore if tertiary education may mediate or moderate the subsequent labor market outcome.

## Methods

### Study population and design

Participants were members of the Uppsala Longitudinal Adolescent Depression Study (ULADS), a Swedish community-based cohort prospectively followed from adolescence onwards [32]. Initially, a total population of first-year students aged 16–17 in high school were enrolled in an epidemiologic investigation in 1991–1992, including same-aged school dropouts [33]. In all, 2300 (93% participation rate) underwent screening by means of two self-report questionnaires: the Beck Depression Inventory—Child (BDI-C) [34] and the Center for Epidemiological Studies—Depression Scale for Children (CES-DC) [35]. Adolescents with positive screening, defined as BDI-C ≥ 16 or CES-DC ≥ 30 + BDI-C ≥ 11 or a self-reported suicide attempt, were invited to take part in a comprehensive face-to-face assessment, including a blinded structured diagnostic interview and additional self-report questionnaires. An equal number of adolescents with negative screening, matched for gender, age, and school class, were also invited. Out of 710 selected adolescents, 631 (78% females) participated. After approximately 15 years, all eligible cohort members were contacted again, of which 409 (65%) were followed up with comprehensive reassessments.

Extensive deidentified data were retrieved in 2017–2018 through linkage of the retained cohort members’ unique personal identity number to various nationwide population-based registries kept by government agencies [22]. This present registry-based follow-up harvested registry data for 576 (91%) of the original cohort members, excluding 55 individuals who at baseline did not give consent to further participation (*n* = 22), or who subsequently had refused extraction of individualized registry data (*n* = 33). Out of the retained cohort (*n* = 576), only those presenting with a hypomanic or manic episode in adolescence (*n* = 37) were excluded for the present study. The rationale for this was that bipolar disorders are classified separately from depressive disorders in the Diagnostic and Statistical Manual of Mental Disorders (DSM) [36], due to key differences in symptomology and etiology. This left a total of 539 eligible cohort members (*n* = 321 with adolescent depression; *n* = 218 with no adolescent depression).

Further details about the data collection waves of the ULADS have been described elsewhere [32]. Ethical approval for the registry-based follow-up was obtained from the Regional Ethical Review Board in Uppsala (2015/449/1-2).

### Measures

#### Exposure

##### Adolescent depression

At baseline, cohort members were assessed with the Diagnostic Interview for Children and Adolescents in the Revised form according to DSM-III-R for Adolescents (DICA-R-A) covering a wide range of childhood and adolescent psychiatric disorders [37]. As the diagnostic assessments were based on DSM-III-R criteria [38], these original classifications were converted to the current DSM-5 taxonomy [36]. The following subgroups were identified:

1. *Persistent depressive disorder (PDD)*: Depressed mood occurring for most of the day, for more days than not, for at least one year (*n* = 175);
2. *Episodic major depressive disorder (MDD)*: A current or life-time major depressive episode lasting shorter than 1 year (*n* = 82);
3. *Subthreshold depression*: A positive screening but no other past or current depressive disorder (*n* = 64);
4. *No depression*: Negative screening, no past or current depressive disorder (*n* = 218).

#### Labor market outcomes

Registry-based data from the Longitudinal Integration Database for Health Insurance and Labor Market Studies (LISA) [22], held by Statistics Sweden, were used as outcome variables. These registry data spanned from early (age 21 or 22) to middle (age 40 or 41) adulthood (i.e., 1997–2016). The LISA registry is updated each year by the transmission of annual data on the labor market, educational systems, and social sectors from various source registries. The database includes all individuals (aged ≥ 16) who were registered as residents in Sweden as of December 31 each year.

Four indicators of LMM were used as outcomes: (1) long-term unemployment, (2) work disability, (3) social welfare assistance, and (4) a composite LMM measure.

Long-term unemployment was defined as ≥ 180 annual net days registered as full- or part-time unemployed or as included in labor market programs at the Swedish Public Employment Service, in line with prior research [39, 40]. To qualify for unemployment benefits, the individual needs to be unemployed, able to work and ready to accept work that is offered. Also, the beneficiary must have worked for ≥ 6 months prior to becoming unemployed.

Work disability was defined as granted disability pension or ≥ 60 annual net days of sickness absence, which built upon official classifications used by national authorities [41] and prior research [11]. In Sweden, all residents aged ≥ 16 years who have an income from work or unemployment benefits are eligible for sickness benefits, provided that there is a reduced work capacity due to disease or injury. For the first 14 days of a sickness period, an employee receives sick pay from the employer, though there is one qualifying day (more for self-employed) without any benefits. If the work capacity is still reduced after 14 days, the employee can apply for sickness cash benefits and, if approved, it is only these exceeding sick leave days that are registered at the Swedish Social Insurance Agency.

Social welfare assistance was defined as a receipt of public assistance, regardless of monetary amount. In Sweden, this is a cash income allowance from the local social authorities, which is given only after a thorough individual means test to guarantee a minimum standard of living [22]. This allowance is designed to cover basic expenditures, such as food and rent, and applies to all residents provided that they try to achieve economic self-sufficiency (i.e., job seeking if unemployed).

Composite LMM was defined as an occurrence of any type of marginalization, including long-term unemployment, work disability, and social welfare assistance.

#### Adult depression

Data from the 15-year follow-up study were used to classify occurrences of any major depressive episodes from age 19 to around 31, as assessed with the M.I.N.I. Plus [42] and a life-chart. Depression in early adulthood was defined as either one long episode (≥ 6 months) or recurrent episodes (≥ 2), as reported in previous work [20, 25, 26], and was thus a categorical variable.

#### Tertiary education

Registry-based information from the LISA database was used to classify the level of education [22]. Two categorical variables were used to reflect educational aspiration and educational attainment, respectively. The former included entry into tertiary education up to age 25/26, whereas the latter had a cut-off of ≥ 3 years of completed tertiary education by age 30/31. As such, those obtaining a degree/diploma were grouped together with those completing at least 3 years of tertiary education, to capture a contextually relevant measure of educational attainment. Importantly, the Swedish official statistics show considerable variation in graduation rates across various educational programs, and an academic degree/diploma is not necessarily required for obtaining employment in several areas of the labor market (e.g., engineering, school teaching) [43].

#### Covariates

##### Individual characteristics

In line with what has been suggested in recent meta-analytic work [3], a range of potential confounding factors were taken into account, including childhood/adolescent anxiety disorder (separation anxiety, overanxious, and avoidant disorder) [44], disruptive behavior disorder (oppositional defiant disorder, conduct disorder, and attention-deficit/hyperactivity disorder) [45], and tobacco use/misuse [46, 47]. These conditions, assessed at baseline using DICA-R-A [37], were included as separate categorical variables (e.g., anxiety disorder, yes/no). Further, gender was included as a categorical variable [3]. Information on grade point average (GPA) from the final year of compulsory school, retrieved from the Pupil Registry, was included as a continuous variable (range 1.0–5.0), as a proxy measure of cognitive ability [48, 49].

##### Family socioeconomic background

Information on parental socioeconomic conditions in the year 1990 was retrieved from the Education Registry, and the Registry of Income and Taxation [22], to account for potential confounding by childhood socioeconomic status [3]. The highest level of education achieved by either parent was included as a categorical variable (i.e., tertiary education, yes/no). The highest disposable income of either parent was assigned to predefined quintiles as determined by the earnings distribution of a contemporaneous reference population (*N* > 200,000), and then dichotomized into a categorical variable (i.e., earnings within or above the 3rd quintile, yes/no).

### Statistical analyses

All LMM indicators were based on consecutive annual data, and each indicator was dichotomized conditional on its occurrence in each year spanning from 1997 to 2016 (i.e., 20 years). Longitudinal associations were examined using logistic regression models in a generalized estimating equations framework [50], including an auto-regressive working correlation structure and robust standard errors, chosen based on model fit. These models were used to look at differences in adult LMM outcomes by adolescent depression status, with the nondepressed peers as the reference category throughout all analyses. To test whether the association between adolescent depression and adult LMM outcomes vary by gender, the models were extended to include interaction terms along with tests of main effects. Potential gender differences in adult LMM outcomes were also examined in stratified analysis. Any between-groups difference was estimated as an odds ratio (OR) with a 95% confidence interval (CI), and statistical significance was set at *p* < 0.05.

The indirect effect of depression in early adulthood (ages 19–30) on the subsequent LMM outcome (ages 31–40) was tested in mediation analysis based on the counterfactual framework for causal inference [51]. Analyses were performed using a logistic regression model for the mediator (i.e., adult depression; binary variable, yes/no) and a quasi-Poisson regression model for the outcome (i.e., subsequent LMM; count outcome, frequency of years in any marginalization), with robust standard errors. Likewise, educational attainment by age 30 was explored as a putative mediator for the subsequent LMM outcome using the same modeling approach.

Effect modification by entry into tertiary education at age 25/26 was explored using interaction terms between adolescent depression status and educational aspiration, alongside tests of main effects. Interaction terms were thus included to test if there are any differences in the direction or strength of the association with the adult LMM outcome. An interaction effect between adolescent depression status and entry into tertiary education would imply potentially important heterogeneity. In terms of the temporal ordering of events, the effect of entry into tertiary education on the subsequent risk for LMM was explored for the latter 15 years of the follow-up period. Potential differences in the subsequent risk for LMM were also examined in stratified analysis.

Data management and analyses were performed using IBM SPSS Statistics version 26 and R version 4.0.3. The mediation analyses were performed using the package 'mediation' [52].

### Missing data

Across all 20 years of follow-up, 10,214 (94.7%) observations were recorded for each LMM outcome. There were two sources of complete or intermittent missingness in the registry-based data: death (*n* = 3) and resettling abroad (*n* = 65). The mean total time residing abroad among those who had emigrated was 7.4 years (SD = 5.9). The overall drop-out rate did not differ substantially between depressed and nondepressed cohort members. However, those with subthreshold depression were more likely to drop out than the other subgroups [32]. All statistical analyses made use of all available follow-up data, given the relatively small proportion of missingness for the main analyses. The analytic sample for the mediation models addressing the indirect effect of adult depression on the subsequent LMM outcome was limited to the subset of cohort members who participated both in the 15-year follow-up and in the present registry-based follow-up (*n* = 351), while the models for the indirect effect of educational attainment was based on all cohort members retained for the study.

## Results

Key characteristics of the cohort members are summarized in Table 1. About four of five in the included cohort were females. Among those with adolescent depression (*n* = 321), the majority of cases presented with PDD (*n* = 175, 54.5%). Moreover, 40 percent of those with adolescent depression had entered tertiary education by age 25/26, as compared with 49 percent among those with no adolescent depression. Thirty-six percent of those with adolescent depression had completed at least 3 years of tertiary education by age 30/31, compared with 43 percent among those with no adolescent depression. The average frequency of years meeting cut-off for the composite LMM outcome across ages 21 to 40 was 4.0 years (SD = 4.7) and 2.2 years (SD = 3.7) among the depressed and the nondepressed groups, respectively.

**Table 1:** Descriptives

Key characteristics	Included cohort (*n* = 539)	No depression (*n* = 218)	Adolescent depression (*n* = 321)	Type of adolescent depression		
				Subthreshold depression (*n* = 64)	Major depressive disorder (*n* = 82)	Persistent depressive disorder (*n* = 175)
	*n* (%)	*n* (%)	*n* (%)	*n* (%)	*n* (%)	*n* (%)
Females	425 (79%)	171 (78%)	254 (79%)	46 (72%)	68 (83%)	140 (80%)
Anxiety disorders^a^	177 (33%)	32 (15%)	145 (45%)	6 (9%)	34 (41%)	105 (60%)
Disruptive behavior disorders^a^	112 (21%)	16 (7%)	96 (30%)	16 (25%)	20 (24%)	60 (34%)
Tobacco use/misuse^a^	227 (42%)	57 (26%)	170 (53%)	37 (58%)	42 (52%)	91 (52%)
Grade point average^b^, M (SD)	3.3 (0.7)	3.5 (0.6)	3.3 (0.7)	3.3 (0.7)	3.2 (0.8)	3.3 (0.6)
Low parental education^c^	254 (49%)	102 (47%)	152 (50%)	30 (51%)	45 (58%)	77 (45%)
Low parental income^d^	177 (34%)	68 (32%)	109 (35%)	20 (33%)	27 (35%)	62 (36%)
Reassessment at 15-year follow-up	351 (65%)	147 (67%)	204 (64%)	30 (47%)	59 (72%)	115 (66%)
Depression in early adulthood^e^						
Yes	134 (38%)	31 (21%)	103 (51%)	11 (37%)	21 (36%)	71 (62%)
No	217 (62%)	116 (79%)	101 (49%)	19 (63%)	38 (64%)	44 (38%)
Entry into tertiary education^f^						
Yes	235 (44%)	107 (49%)	128 (40%)	25 (39%)	33 (41%)	70 (40%)
No	303 (56%)	111 (51%)	192 (60%)	39 (61%)	48 (59%)	105 (60%)
Educational attainment^g^						
≥ 3 years	208 (39%)	93 (43%)	115 (36%)	23 (36%)	29 (36%)	63 (36%)
No or < 3 years	330 (61%)	125 (57%)	205 (64%)	41 (64%)	52 (64%)	112 (64%)
Labor market outcome^h^						
Long-term unemployment, M (SD)	1.8 (2.8)	1.3 (2.3)	2.1 (3.0)	1.6 (2.5)	1.9 (2.4)	2.4 (3.5)
Work disability, M (SD)	1.2 (2.8)	0.8 (2.5)	1.4 (3.0)	0.8 (1.6)	1.3 (2.8)	1.7 (3.4)
Social welfare recipiency, M (SD)	0.9 (2.3)	0.4 (1.3)	1.2 (2.7)	1.3 (2.7)	0.9 (2.5)	1.3 (2.8)
Composite measure, M (SD)	3.3 (4.4)	2.2 (3.7)	4.0 (4.7)	3.0 (3.8)	3.5 (4.3)	4.6 (5.2)

^a^In childhood/adolescence

^b^Academic performance in the 9th school year (*n* = 530)

^c^Neither parent had tertiary education (*n* = 522)

^d^The two lowest quintiles of the income distribution (*n* = 525)

^e^Ages 19–30

^f^Enrollment in tertiary education by age 25 (*n* = 538)

^g^Level of tertiary education by age 30 (*n* = 538)

^h^Number of years meeting cut-off across ages 21–40

### Associations of adolescent depression with adult labor market outcomes

Compared with nondepressed peers, adolescents with depression had significantly higher odds of all LMM outcomes in unadjusted analyses, as shown in Table 2. After covariate adjustment, the associations with long-term unemployment (aOR = 1.2, 95% CI = 0.9–1.6, *p* = 0.219) and work disability (aOR = 1.4, 95% CI = 0.9–2.3, *p* = 0.142) became nonsignificant, while the associations with social welfare assistance (aOR = 2.1, 95% CI = 1.3–3.5, *p* = 0.004) and the composite outcome (aOR = 1.5, 95% CI = 1.1–2.0, *p* = 0.005) attenuated but remained significant.

**Table 2:** Longitudinal associations between adolescent depression and adult labor market outcome across ages 21–40

Labor market outcome	No depression (*n* = 218)	Adolescent depression (*n* = 321)		Significant covariates
		Unadjusted	Adjusted^a^	
		OR (95% CI)	OR (95% CI)	
Long-term unemployment	Ref.	1.7*** (1.3–2.3)	1.2 (0.9–1.6)	5, 7
Work disability	Ref.	1.8* (1.1–2.9)	1.4 (0.9–2.3)	–
Social welfare assistance	Ref.	3.3*** (2.0–5.5)	2.1** (1.3–3.5)	3, 5
Composite measure	Ref.	2.1*** (1.6–2.8)	1.5** (1.1–2.0)	3, 5, 7

*OR* odds ratio, *CI* confidence interval

*p < 0.05, **p < 0.01, ***p < 0.001

^a^Simultaneous adjustment for all covariates, including: 1 = gender; 2 = anxiety disorders; 3 = disruptive behavior disorders; 4 = tobacco use/misuse; 5 = grade point average; 6 = parental education; 7 = parental income

As presented in Table 3, those with PDD had significantly higher odds of all LMM outcomes throughout both unadjusted and adjusted analyses, including elevated long-term unemployment (aOR = 1.5, 95% CI = 1.0–2.1, *p* = 0.034), work disability (aOR = 1.8, 95% CI = 1.1–3.1, *p* = 0.021), social welfare assistance (aOR = 3.0, 95% CI = 1.7–5.3, *p* < 0.001), and the composite LMM outcome (aOR = 2.0, 95% CI = 1.4–2.7, *p* < 0.001). Although those with episodic MDD and subthreshold depression also had significantly higher odds of some LMM outcomes, none of these associations held after adjustment for covariates.

**Table 3:** Longitudinal associations between adolescent depression subcategories and adult labor market outcome across ages 21–40

Labor market outcome	No depression (*n* = 218)	Subthreshold depression (n = 64)		Major depressive disorder (*n* = 82)		Persistent depressive disorder (*n* = 175)		Significant covariates
		Unadjusted	Adjusted^a^	Unadjusted	Adjusted^a^	Unadjusted	Adjusted^a^	
		OR (95% CI)	OR (95% CI)	OR (95% CI)	OR (95% CI)	OR (95% CI)	OR (95% CI)	
Long-term unemployment	Ref.	1.2 (0.8–2.0)	0.9 (0.6–1.3)	1.6* (1.1–2.3)	1.1 (0.7–1.6)	2.0*** (1.4–2.8)	1.5* (1.0–2.1)	5, 7
Work disability	Ref.	0.9 (0.5–1.8)	0.8 (0.4–1.7)	1.7 (0.9–3.2)	1.4 (0.7–2.7)	2.2** (1.3–3.7)	1.8* (1.1–3.1)	–
Social welfare assistance	Ref.	3.4*** (1.7–6.7)	1.5 (0.8–2.9)	2.4* (1.2–5.1)	1.5 (0.7–3.2)	3.6*** (2.1–6.2)	3.0*** (1.7–5.3)	3, 5
Composite measure	Ref.	1.5 (1.0–2.3)	1.0 (0.7–1.5)	1.8** (1.2–2.7)	1.3 (0.9–2.0)	2.5*** (1.8–3.4)	2.0*** (1.4–2.7)	3, 5, 7

*OR* odds ratio, *CI* confidence interval

*p < 0.05, **p < 0.01, ***p < 0.001

^a^Simultaneous adjustment for all covariates, including: 1 = gender; 2 = anxiety disorders; 3 = disruptive behavior disorders; 4 = tobacco use/misuse; 5 = grade point average; 6 = parental education; 7 = parental income

### Differences by gender

As for differential outcomes by gender, the results showed a significant main effect of adolescent depression (OR = 1.5, 95% CI = 1.1–2.0, *p* = 0.021), a nonsignificant main effect of male gender (OR = 0.5, 95% CI = 0.3–1.0, *p* = 0.064), and a significant group-by-gender interaction (OR = 2.4, 95% CI = 1.1–5.3, *p* = 0.030) with regard to long-term unemployment. There were no significant interactions with other outcomes, nor was gender a significant predictor in other analyses (results not shown).

As presented in Table 4, stratified analyses confirmed that males with adolescent depression were at increased odds of long-term unemployment; however, this association attenuated and became nonsignificant after covariate adjustment (aOR = 1.7, 95% CI = 0.8–3.7, *p* = 0.186). Similarly, females with adolescent depression were found to be at increased odds of work disability, although this association attenuated slightly and became nonsignificant after covariate adjustment (aOR = 1.6, 95% CI = 1.0–2.7, *p* = 0.060). Even after adjustment, however, the depressed females had significantly higher odds of social welfare assistance (aOR = 2.2, 95% CI = 1.3–3.7, *p* = 0.004) and the composite LMM outcome (aOR = 1.5, 95% CI = 1.1–2.0, *p* = 0.007). For both these latter outcomes, however, nonsignificant associations of similar magnitudes were also observed among the depressed males.

**Table 4:** Gender-stratified longitudinal associations between adolescent depression and adult labor market outcome across ages 21–40, with nondepressed controls as reference

Labor market outcome	Adolescent depression (*n* = 321)		Significant covariates
	Unadjusted	Adjusted^a^	
	OR (95% CI)	OR (95% CI)	
Long-term unemployment			
Females	1.5* (1.1–2.0)	1.1 (0.8–1.5)	4, 6
Males	3.5*** (1.7–7.1)	1.7 (0.8–3.7)	1
Work disability			
Females	2.0** (1.2–3.2)	1.6 (1.0–2.7)	6
Males	1.2 (0.3–4.1)	0.9 (0.3–2.8)	–
Social welfare assistance			
Females	3.2*** (1.8–5.7)	2.2** (1.3–3.7)	2, 4
Males	3.6* (1.3–10.6)	2.0 (0.7–6.0)	–
Composite measure			
Females	1.9*** (1.4–2.7)	1.5** (1.1–2.0)	4, 6
Males	2.7** (1.3–5.4)	1.5 (0.8–3.1)	1

*OR* odds ratio, *CI* confidence interval

*p < 0.05, **p < 0.01, ***p < 0.001

^a^Simultaneous adjustment for all covariates, including: 1 = anxiety disorders; 2 = disruptive behavior disorders; 3 = tobacco use/misuse; 4 = grade point average; 5 = parental education; 6 = parental income

### The indirect effect of adult depression

Depression in early adulthood was significantly associated with the subsequent LMM outcome, both in unadjusted (incidence rate ratio = 2.4, 95% CI = 1.8–3.2, *p* < 0.001) and adjusted analysis (incidence rate ratio = 2.3, 95% CI = 1.7–3.3, *p* < 0.001), where the latter included prior exposure to adolescent depression along with all aforementioned covariates.

As shown in Table 5, the unadjusted analysis revealed a significant indirect effect of adult depression (*B* = 0.25, 95% CI = 0.09–0.46, *p* < 0.001) along with a significant direct effect of adolescent depression (*B* = 0.56, 95% CI = 0.11–1.00, *p* = 0.014) with regard to subsequent LMM. After covariate adjustment, the direct effect of adolescent depression attenuated and became nonsignificant whereas the indirect effect remained significant. The average proportion mediated after adjustment for covariates was 35 percent.

**Table 5:** The mediating effect of recurrent depression in early adulthood (ages 19–30 years) on the subsequent labor market outcome in mid-adulthood (ages 31–40) among those with adolescent depression, with nondepressed controls as reference

Depression status in adolescence	Depression in early adulthood^a^		Ratio of years in overall labor market marginalization^b^			Proportion mediated (average)
	Proportion (95% CI)	OR (95% CI)	Total effect *B* (95% CI)	Average direct effect (average) *B* (95% CI)	Average causal mediation effect (average) *B* (95% CI)	
Unadjusted						
No depression (*n* = 147)	0.21 (0.14–0.28)	–	–	–	–	0.30***
Adolescent depression (*n* = 204)	0.50 (0.44–0.57)	3.8*** (2.4–6.2)	0.82*** (0.38–1.27)	0.56* (0.11–1.00)	0.25*** (0.09–0.46)	
Adjusted^c^						
No depression (*n* = 147)	0.21 (0.14–0.28)	–	–	–	–	0.35*
Adolescent depression (*n* = 204)	0.51 (0.44–0.58)	2.8*** (1.6–4.8)	0.55* (0.10–1.06)	0.35 (-0.09–0.84)	0.20*** (0.06–0.39)	

*OR* odds ratio, *CI* confidence interval

*p < 0.05, **p < 0.01, ***p < 0.001

^a^Ages 19–30

^b^Ages 31–40

^c^Simultaneous adjustment for all covariates, including: 1 = gender; 2 = anxiety disorders; 3 = disruptive behavior disorders; 4 = tobacco use/misuse; 5 = grade point average; 6 = parental education; 7 = parental income

As presented in Table 6, for those with PDD in adolescence, the unadjusted analysis showed an indirect effect of adult depression (B = 0.36, 95% CI = 0.11–0.68, *p* < 0.001) and a direct effect of PDD (*B* = 0.92, 95% CI = 0.34–1.56, *p* < 0.001), and these estimates remained roughly unchanged in the adjusted analysis. The average proportion mediated after covariate adjustment was 31 percent.

**Table 6:** The mediating effect of recurrent depression in early adulthood (ages 19–30 years) on the subsequent labor market outcome in mid-adulthood (ages 31–40) among those with adolescent persistent depressive disorder, with nondepressed controls as reference

Depression status in adolescence	Depression in early adulthood^a^		Ratio of years in overall labor market marginalization^b^			Proportion mediated (average)
	Proportion (95% CI)	OR (95% CI)	Total effect *B* (95% CI)	Average direct effect (average) *B* (95% CI)	Average causal mediation effect (average) *B* (95% CI)	
Unadjusted						
No depression (*n* = 147)	0.21 (0.14–0.28)	–	–	–	–	0.28***
Persistent depressive disorder (*n* = 115)	0.62 (0.53–0.71)	6.0*** (3.5–10.4)	1.29*** (0.72–1.96)	0.92*** (0.34–1.56)	0.36*** (0.11–0.68)	
Adjusted^c^						
No depression (*n* = 147)	0.21 (0.14–0.28)	–	–	–	–	0.31**
Persistent depressive disorder (*n* = 115)	0.61 (0.52–0.70)	4.5*** (2.3–8.7)	1.08** (0.42–1.86)	0.74* (0.09–1.51)	0.34*** (0.10–0.67)	

*OR* odds ratio, *CI* confidence interval

*p < 0.05, **p < 0.01, ***p < 0.001

^a^Ages 19–30

^b^Ages 31–40

^c^Simultaneous adjustment for all covariates, including: 1 = gender; 2 = anxiety disorders; 3 = disruptive behavior disorders; 4 = tobacco use/misuse; 5 = grade point average; 6 = parental education; 7 = parental income

Regarding the MDD and subthreshold subgroups, there was no significant indirect effect of adult depression on the subsequent LMM outcome (results not shown).

### The role of tertiary education

As regards, the composite LMM outcome across ages 26–40, the moderation analysis yielded significant main effects of adolescent depression in general (OR = 1.8, 95% CI = 1.0–3.2, *p* = 0.037) and no entry into tertiary education (OR = 1.9, 95% CI = 1.0–3.3, *p* = 0.036), but no evidence of any significant interaction effect between adolescent depression and entry into tertiary education. However, in the subgroup analysis including the various clinical forms of adolescent depression, there were significant main effects of PDD (OR = 2.7, 95% CI = 1.5–4.9, *p* = 0.002), and no entry into tertiary education (OR = 1.8, 95% CI = 1.0–3.3, *p* = 0.038), and also a significant interaction effect for episodic MDD and entry into tertiary education (OR = 3.1, 95% CI = 1.2–8.5, *p* = 0.024).

As shown in Table 7, stratified analysis confirmed that there was a significant unadjusted association between episodic MDD and LMM among those with no entry into tertiary education (OR = 2.2, 95% CI = 1.3–3.7, *p* = 0.005), but not among those who entered tertiary education (OR = 0.7, 95% CI = 0.3–1.5, *p* = 0.355). These associations attenuated slightly after covariate adjustment, but remained significant. Moreover, the higher odds of LMM was again observed among those with PDD, regardless of educational paths.

**Table 7:** Overall labor market outcome across ages 26–40, stratified by entry into tertiary education ﻿a﻿﻿﻿t age approximately 25

Variables	Composite measure of labor market marginalization			
	No tertiary education (*n* = 303)		Tertiary education (*n* = 235)	
	Unadjusted	Adjusted^a^	Unadjusted	Adjusted^a^
	OR (95% CI)	OR (95% CI)	OR (95% CI)	OR (95% CI)
Type of adolescent depression				
No depression (Ref.)	–	–	–	–
Subthreshold depression	1.3 (0.7–2.3)	0.9 (0.5–1.7)	1.3 (0.5–2.9)	0.7 (0.3–1.8)
Major depressive disorder	2.2** (1.3–3.7)	1.8* (1.0–3.0)	0.7 (0.3–1.5)	0.6 (0.2–1.5)
Persistent depressive disorder	2.2*** (1.4–3.6)	2.0** (1.2–3.3)	2.7** **(1.5–4.9)**	1.8* (1.0–3.2)
Gender				
Female	–	1.5 (0.9–2.6)	–	0.9 (0.3–2.3)
Individual characteristics				
Anxiety disorders	–	1.0 (0.7–1.5)	–	1.1 (0.6–2.0)
Disruptive behavior disorders	–	1.2 (0.8–2.0)	–	3.4*** (1.6–7.2)
Tobacco use/misuse	–	0.9 (0.6–1.4)	–	1.1 (0.6–2.2)
Grade point average	–	0.5*** (0.3–0.7)	–	1.4 (0.8–2.5)
Family socioeconomic background				
Low parental education	–	0.7 (0.4–1.0)	–	0.9 (0.4–1.9)
Low parental income	–	1.4 (0.9–2.0)	–	1.6 (0.9–3.1)

*OR* odds ratio, *CI* confidence interval

*p < 0.05, **p < 0.01, ***p < 0.001

^a^Simultaneous adjustment for all included covariates

There was no evidence of any indirect (mediating) effects of educational attainment by age 30 with respect to the subsequent LMM outcome across ages 31–40. This was the case both for the broader category of depressed adolescents and for the breakdown of subgroups (results not shown).

## Discussion

This longitudinal study examined important labor market outcomes following adolescent depression based on a diagnostically well-characterized and community-representative cohort prospectively followed over more than 2 decades. The study advances our understanding of the longer-term outcome of early-life depression, as we attempted to capture previously overlooked sources of heterogeneity. Consistent with prior research, we found that the associations between adolescent depression and adult LMM outcomes attenuated after adjustment for potential confounders. Further, there was a somewhat differential outcome by gender ;compared to their nondepressed counterparts, the depressed males had much higher odds of long-term unemployment while the depressed females had higher odds of work disability. These observations may, in part, be explained by confounding factors such as psychiatric comorbidity and family adversity. In terms of clinical differentiation of depression, we found that adolescents with PDD had markedly higher odds of all LMM outcomes, and that these associations were generally robust to adjustment for various markers of risk. There were less conclusive results regarding the overall risk for LMM among those with episodic MDD and subthreshold depression, even though a similar direction of findings was partly observed in these subgroups. Moreover, mediation analyses confirmed an indirect effect, suggesting that the longitudinal link between adolescent depression and adult LMM may in part be explained by subsequent depression recurrence in early adulthood. This link was found to be mainly driven by those presenting with PDD in adolescence, in line with our previous findings [20, 26]. Further, while there was no evidence of an indirect effect of educational attainment, the exploratory moderation analyses suggested that the effect of episodic MDD on the subsequent risk for LMM seem to be mitigated for those enrolling in tertiary education in early adulthood. However, entry into tertiary education was not differentially associated with LMM for other forms of adolescent depression.

This study had the opportunity to expand upon prior research by addressing important questions revolving around adult labor market outcomes following adolescent depression. The study adds to recent work on longer-term outcomes, suggesting that the effects of early chronic/persistent depression extend into adulthood and interfere with critical domains of psychosocial functioning [19, 20]. The inconclusive pattern of results regarding episodic MDD and subthreshold depression raises the long-standing question whether early nonchronic depression is more of a transient developmental feature for certain individuals, or perhaps have a more lasting impact on only some adult life domains [53]. Furthermore, in this study, the rates of entry into tertiary education by the mid-twenties were found to be strikingly similar across the depressed subgroups (around 40 percent), but somewhat lower than among the nondepressed peers, of whom nearly half had enrolled in tertiary level education. This finding resonates well with recent meta-analytic evidence suggesting that adolescent depression is associated with lower odds of entry into tertiary education, as compared with nondepressed controls [3].

In terms of potential gender differences in LMM, our results were suggestive of a somewhat divergent outcome. Exposure to adolescent depression was associated with a substantially elevated risk of long-term unemployment among the males, although this association attenuated considerably after adjustment for confounders. While the vast majority of previous long-term follow-up studies have failed to report results separately for both genders, thereby making comparisons across studies difficult, this finding is generally in keeping with some observations [13, 39, 54], but at odds with other studies suggesting that depressed females might be worse off [16, 55]. Other follow-up studies have found no clear evidence of gender-specific associations [17, 19, 23]. These inconsistent findings may either imply that the question remains unsettled, possibly due to methodological discrepancies between studies, or perhaps that the relevant social/historical context may have a differential impact on the employment prospects of males and females with early-life depression. Further, our results indicated that females with early depression had increased risk of work disability. This finding adds to a recent official report showing that working-age women are at a particularly high risk of sick leave due to psychiatric disorders [56]. While the causative factors behind the widely reported gender gap in sickness absence remain unclear, and likely complex, it has been suggested that various factors such as stressful life events, work-life conflict, and labor market conditions might be important contributors [57–63]. Although our adjusted analyses indicated that the associations were partly confounded by individual characteristics as well as family socioeconomic background, these results may nonetheless suggest potentially different selection mechanisms or routes into adult marginalization among males and females with prior depression.

Importantly, the developmental pathways linking adolescent depression to adverse adult health and functioning are still largely unclear [17], as there has been surprisingly little research into the mechanisms of the prospective outcome. The often chronic or recurring course of the disorder emerges as a plausible explanation to various adversities and hardships in adulthood, including mental ill-health, general medical conditions, and economic disadvantage, as reported in our prior work [20, 25, 26]. For example, we found that the association between adolescent PDD and adulthood earnings was partially mediated (around 50 percent) by recurrent episodes of depression in early adulthood [20]. More broadly, early exposure to depression or depression symptoms may also interfere with normal development in other important life domains, such as school performance (e.g., GPA) [64] and attainment of tertiary education [3], which are important factors in the context of labor market outcomes [13]. While previous research has indicated that educational attainment [16] and recurring bouts of depression [16, 20] may play an intermediate role in determining adult socioeconomic outcome, there has been no study to date addressing the risk of marginalization from the labor market while accounting for various sources of important heterogeneity associated with adolescent depression and its longer-term outcomes. In the present study, we sought to expand on the preliminary evidence pointing to the roles played by both the educational paths taken and the illness course of depression. The results provide evidence to suggest that depression recurrence extending into early adulthood partially explains the elevated risk of marginalization from the labor market, especially for those with PDD in adolescence. Moreover, the results also suggest that entry into tertiary education may be protective against marginalization from the labor market, but perhaps only for those with episodic MDD, implying that those with PDD are at increased risk of later marginalization regardless of educational aspiration and attainment. As already noted, one plausible mechanism for the elevated risk of marginalization from the labor market might lie with the more severe course of illness following early-onset PDD [2, 20]. However, more rigorous large-scale research is needed to better understand the underlying determinants and selection processes for education and labor market performance, not least because unobserved heterogeneity and unmeasured familial confounders might be at play [65].

Recent longitudinal research suggests that the linkages between adolescent depression and adverse adult outcomes are mainly attributable to individual, familial, and social factors, rather than depression per se [17]. The present study challenges this notion and contributes with additional information about the relevance of clinical differentiation of adolescent depression with respect to adult life outcomes. While milder forms of depression may not be independently related to later difficulties in the labor market, there is now growing evidence to substantiate the unfavorable life prospects following early chronic/persistent depression. Previous research linking adolescent depression to pervasive deficits in adult psychosocial functioning suggests that the residual effects of depression may remain in the form of 'scars' exerting a pernicious influence on later development [66]. These long-term scarring effects may be more clustered in those exposed to early chronic/persistent depression as compared with other forms of early depression. Similarly, there is the possibility that the heightened risk of poorer long-term outcomes associated with early chronic/persistent depression can be rooted in psychosocial impairments already existing in childhood or adolescence [67–70], and potentially that these problems may exacerbate over time, which in some cases arguably could be related to the longitudinal course of depressive illness. This points to the need of early identification and intervention targeting adolescents with depression, to mitigate long-lasting impairments.

The overall findings of this study, along with the well-documented poor psychosocial prognosis of adolescent depression [3, 4, 19, 20, 32], underscore the importance of efficacious prevention and early treatment to avert future adversities and related societal costs. Our data support that depression in early life may have a stagnating effect on human capital development and workforce participation [3, 4], particularly for those exposed to early chronic/persistent depression. While future replication is needed, it is likely to be a worthy investment to counteract this negative trend early on through scaling up healthcare services for depression [71]. To better inform policymaking, the impact of scaling up early targeted interventions should ideally be addressed in future research using prospective pragmatic cohort studies. Interestingly, several innovative programs and services have already been developed to help psychologically distressed youths at risk of long-term economic disadvantage and marginalization from the labor market, with promising results in terms of positive returns on investment [72], further affirming the importance of timely and targeted interventions.

Some strengths of this study are particularly noteworthy. The study utilized a distinct combination of data from in-depth diagnostic interviews, self-report questionnaires, and nationwide population-based registries, allowing for novel analyses of relevant labor market outcomes based on a community-representative sample. Notably, this work emphasizes the relevance of diagnostic differentiation between clinical forms of early depression, in particular with respect to PDD and episodic MDD. The integration of more than 2 decades of longitudinal data, and a high retention rate withal, provides a more complete estimation of the labor market outcomes associated with adolescent depression than previously reported. Few other prospective cohort studies on adolescent depression have included such extensive and objective measures of marginalization. However, the findings should be interpreted in the context of some limitations. First, unmeasured factors including genetic vulnerability may account for observed associations [73–75], and other forms of residual confounding cannot be ruled out. Second, the study was not without attrition, yet the study retained around 90 percent of the original cohort, with a relatively small proportion of missingness across the follow-up period. Third, there was no differentiation between childhood- or adolescence-onset of depression, precluding any further tests of outcomes predicted by age of onset. Fourth, the statistically nonsignificant associations observed for the various subgroups should be interpreted with caution, as these results may be attributable to a lack of power to detect potential differences. Fifth, the analytic strategy did not permit to examine potential gender differences linked to the clinical forms of adolescent depression, but only adolescent depression more generally, due to the modest cell counts in some of the male subgroups. Sixth, early depression shows not only homotypic continuity over time, but also heterotypic continuity with other psychiatric disorders [76], some of which are widely reported to have adverse effects on employment status and other labor market outcomes as well [77–80]. Early depression has also been found to predict later physical health problems [1], and it seems likely that the depressed adolescents' vulnerability to poor health in general may have impacted on their longer-term global functioning. Finally, the generalizability of results might be restricted by the social and historical context relevant to this particular cohort. Indeed, these overall results align well with previous findings on long-term psychosocial outcomes, but the apparent differences in welfare state architecture across Western countries may be relevant to acknowledge for an improved understanding of future life chances following depression in early life.

In conclusion, this study adds to current evidence linking depression in adolescence to compromised psychosocial functioning in adulthood [3]. Exposure to depression in early life increases the risk of adverse labor market outcomes across early to middle adulthood, especially for chronically depressed adolescents. The elevated risk of marginalization from the labor market may, in part, be attributed to the longitudinal course of depression. Yet, there is preliminary evidence to suggest that entry into tertiary education in early adulthood may be protective against adverse effects in the labor market, at least for those presenting with episodic major depression. Further, findings from this study are suggestive of different outcomes by gender; males with a history of adolescent depressive disorder or symptoms may be at elevated risk of long term unemployment while their female counterparts may have heightened susceptibility to long-lasting sickness absence. More large-scale research is however needed to generate deeper insights into the heterogeneity of adolescent depression, with a specific focus on modifiable risk factors relevant to longer-term health and social functioning. Overall, this study highlights the need for timely and targeted interventions to increase autonomous functioning and avert future societal costs.

## Data Availability

National regulations regarding data retrieved from the Swedish registries prevent us from sharing any data openly, due to reasons related to confidentiality and protection of human privacy.
